# Prevalence and biopsychosocial indicators of fatigue in cancer patients

**DOI:** 10.1002/cam4.7293

**Published:** 2024-05-31

**Authors:** Elisabeth L. Zeilinger, Irina Zrnic‐Novakovic, Claudia Oppenauer, Matthäus Fellinger, Matthias Knefel, Matthias Unseld, Theresa Wagner, Simone Lubowitzki, Rupert Bartsch, Sabine Zöchbauer‐Müller, Markus Raderer, Philipp B. Staber, Peter Valent, Alexander Gaiger

**Affiliations:** ^1^ Division of Hematology and Hemostaseology, Department of Medicine I Medical University of Vienna Vienna Austria; ^2^ Department of Clinical Research SBG Academy for Ageing Research, Haus der Barmherzigkeit Vienna Austria; ^3^ Department of Clinical and Health Psychology, Faculty of Psychology University of Vienna Vienna Austria; ^4^ Division of Clinical Psychology, Department of Psychology and Psychodynamics Karl Landsteiner University of Health Sciences Krems Austria; ^5^ Clinical Division of Social Psychiatry, Department of Psychiatry and Psychotherapy Medical University of Vienna Vienna Austria; ^6^ Second Department of Psychiatry and Psychotherapy Clinic Hietzing, Vienna Healthcare Group Vienna Austria; ^7^ Department of Internal Medicine Landesklinikum Baden‐Mödling Baden Austria; ^8^ Division of Oncology, Department of Internal Medicine I Medical University of Vienna Vienna Austria; ^9^ Ludwig Boltzmann Institute for Hematology and Oncology Medical University of Vienna Vienna Austria

**Keywords:** biopsychosocial models, fatigue, mental health, neoplasms, psycho‐oncology

## Abstract

**Introduction:**

Symptoms of cancer‐related fatigue (CRF) can have a significant impact on patients' quality of life and treatment adherence. We aimed to investigate the relationship between CRF and multiple psychosocial and somatic indicators within a large mixed cancer sample.

**Methods:**

In this cross‐sectional study, *N* = 1787 outpatients with cancer were assessed for CRF, pain, anxiety, and depression using validated screening instruments. We further obtained clinical parameters (Hb, CRP, creatinine, leukocytes, ASAT, and ALAT), sociodemographic data (age, gender, income, education level, marital status, parenthood, and living area), and lifestyle factors. Multivariate linear regression models were applied to estimate the impact of each indicator on CRF.

**Results:**

Overall, 90.6% of patients experienced some CRF, with 14.8% experiencing severe CRF. No gender difference was found in the prevalence of CRF. Patients with higher levels of pain, depressive symptoms, and lower Hb levels had significantly higher levels of CRF (*p*_*s*_ <0.001). Lower levels of CRF were observed in patients who had children (*p* = 0.03), had less education (*p* < 0.001), and were physically active for more than 2 h per week before their oncological diagnosis (*p* = 0.014). The latter was only a significant indicator in the male subsample.

**Conclusion:**

The present results demonstrate a high prevalence of CRF and highlight that not only somatic and psychosocial factors, but also lifestyle factors prior to diagnosis appear to be associated with the etiology and persistence of CRF. To effectively treat CRF, a biopsychosocial, personalized approach is recommended.

## 1 INTRODUCTION

Cancer‐related fatigue (CRF) is defined as a subjective feeling of tiredness and lack of energy linked to cancer or cancer treatment. It encompasses physical, cognitive, and emotional exhaustion,^1^ affecting patients' quality of life,^2^ treatment adherence,^3^ and mental health.^4^, ^5^ This fatigue is frequently reported by patients with both solid tumors and hematological cancer,^6^, ^7^ with the former being more commonly studied. CRF has even been associated with reduced survival rates, making it one of the most severe adverse effects of cancer and its treatment.^8^ However, the experience of CRF varies among patients, with prevalence rates ranging from 26% to 62% depending on the cancer type, stage, and treatment phase.^9^

The pathogenesis of fatigue in cancer patients is often characterized as multifactorial.^10^ It has been connected to various metabolic and pathological processes, for example, dysfunction of the hypothalamic–pituitary–adrenal (HPA) activation and the production of pro‐inflammatory cytokines.^11^, ^12^ Inflammatory biomarkers, such as high levels of C‐reactive protein (CRP), are often associated with fatigue and depression in cancer patients, as well as with unresolved and advanced disease in different types of cancer.^13^ Moreover, significant differences in Hb levels between patients with and without CRF were found.^14^ Additional biomarkers linked to CRF include aspartate aminotransferase (ASAT), alanine aminotransferase (ALAT), leukocyte count, and serum creatinine levels.^15^

Apart from biomarkers, fatigue has been related to sociodemographic factors such as gender and education,^9^ which are often referred to as social determinants of health. It is plausible that other social determinants of health, including ethnicity, income, and cumulative stress exposure, might also impact fatigue, as these factors could account for symptom and treatment disparity in earlier studies on cancer patients.^16^ However, it is important to note that some inconsistencies exist with regard to the impact of social determinants of health on CRF. Demographic factors such as age have also been associated with fatigue in some studies,^17^ while other studies have not found this association.^9^ Noteworthy is also a large amount of interindividual variability in fatigue levels identified in previous research.^18^

Recently, increasing attention has been devoted to the psychosocial correlates and lifestyle factors associated with fatigue. By way of illustration, psychosocial interventions (e.g., education about fatigue, coping, and activity management) have proved to be effective in reducing CRF.^19^ A recent umbrella review has also identified mindfulness and cognitive therapies as promising candidates for treating fatigue in cancer patients.^20^ Other modifiable lifestyle factors, particularly physical activity, have repeatedly shown positive effects on fatigue, with physical training being recommended during and after cancer treatment.^18^, ^21^

Furthermore, there is evidence of an association between symptoms of mental health disorders and CRF. Depressive symptoms, sleep disturbances, and distress are the most prevalent factors associated with fatigue,^22^, ^23^ both at the beginning of and throughout the cancer treatment.^24^ Given their high comorbidity and common metabolic processes, the causal pathways between these constructs are not yet completely understood. To illustrate, depression is significantly associated with fatigue symptoms, as well as disease severity and pain,^25^ which have both been linked to fatigue.^26^, ^27^ Moreover, the pattern of change in fatigue over time was altered after adjusting for depressive symptoms in a longitudinal study on women with breast cancer,^28^ highlighting the complexity of the relationship between depression and CRF.

Taken together, numerous psychosocial and somatic indicators have been related to CRF,^29^ but their independent association with fatigue have not yet been fully understood. Especially the psychosocial indicators warrant further investigation, given the increasing evidence about the importance of social determinants of health^2^ and non‐pharmaceutical interventions^30^ in the context of CRF. Moreover, a close examination of prevalence and indicators of CRF across genders and cancer types might contribute to a better understanding of the interindividual variability documented in previous research on CRF.

Therefore, our study aimed to investigate the prevalence of CRF and the relationship between CRF and several psychosocial and somatic indicators in patients with different types of cancer. Additionally, we aimed to account for differences related to gender and cancer type by calculating subanalyses for men and women, and for people with solid tumors and hematological cancers separately.

## 2 MATERIALS AND METHODS

### 2.1 Procedure

Patients receiving cancer treatment at the outpatient unit of oncology and hematology at the Medical University of Vienna were invited to participate if the following inclusion criteria were met: (1) confirmed diagnosis of cancer, (2) age ≥ 18, (3) capacity to consent, (4) sufficient German‐language skills. Written informed consent was obtained from all participants with a 78% response rate. Data were collected by paper and pencil from 2018 to 2023. The study was conducted in accordance with the requirements for Good Clinical Practice outlined in the Declaration of Helsinki and approved by the ethics committee of the Medical University of Vienna (EC Nr: 2255/2016; 1241/2021).

### 2.2 Materials

Data for this study were derived from patient self‐report using a paper and pencil method. Self‐report instruments included visual analog scales (VAS) for fatigue and pain, the Hospital Anxiety and Depression Scale (HADS), a statement about physical activity prior to the cancer diagnosis, and a sociodemographic profile. The clinical data for this study were automatically extracted from the medical records and matched to our data by the hospital's IT system using a patient key.

#### 2.2.1 Assessment of fatigue and pain

Fatigue and pain were each assessed using a VAS with a range of zero to 10. Single‐item screenings are generally a valid and feasible method for assessing fatigue in oncological patients,^31^ and have been shown to be more sensitive than categorical ratings for assessing pain.^32^ The two VASs used in this study each represented a 10 cm line on which the patients indicated their responses. The answers on the VAS were coded using a ruler and rounded to one decimal place for data entry. To describe the present sample, the results of the VAS were grouped into categories, with zero representing no fatigue/pain, one to three points representing mild fatigue/pain, four to six points representing moderate fatigue/pain, and seven to 10 points representing severe fatigue/pain.

#### 2.2.2 Hospital anxiety and depression scale

The HADS is a 14‐item instrument assessing anxiety and depression. All items are rated on a 4‐point Likert scale ranging from zero to three, with higher scores indicating more symptoms. The HADS allows for the calculation of an anxiety score (HADS‐A) and a depression score (HADS‐D), each having a range from 0 to 21. Scores from zero to seven indicate no anxiety/depression, scores from eight to 10 indicate a possible anxiety/depression, and scores higher than 10 indicate significant anxiety/depressive symptoms.^33^ The HADS was shown to have a robust factor structure in mixed cancer samples.^34^ Internal consistencies (Cronbach *α*) in the present study were *α* = 0.85 for HADS‐A, and *α* = 0.87 for HADS‐D.

### 2.3 Statistical method

Fatigue data were log(x + 1)‐transformed due to high skewness. Data transformation has been shown to have superior performance over generalized linear models for right‐skewed data in simulation studies.^35^ First, bivariate associations were analyzed. Second, a multivariate model was computed. Gender differences were explored using chi‐squared tests. For bivariate correlation analysis, we used Pearson's correlation coefficient, point‐biserial correlation coefficient, and phi coefficient, dependent on type of variables. To interpret the correlations, we applied the empirically adapted Cohen's guidelines^36^ for effect sizes,^37^ with *r* ≥ 0.1, *r* ≥ 0.2, and *r* ≥ 0.3 pertaining to a small, medium, and large effect.

For multivariate analysis, we applied linear regression models. Continuous variables entered were pain, anxiety, depression, Hb levels, CRP levels, serum creatinine levels, leukocyte count, ASAT, ALAT, and age. Categorial variables entered were gender (female/male), cancer type (hematologic/solid tumor), marital status (single/in partnership), having children (no/yes), living area (rural/urban), level of education (<12 years/>12 years), income (</> 1300 Euro), and physical exercise prior to the cancer diagnosis (0 h per week/1–2 h per week/>2 h per week). Variance inflation factors (VIFs) indicated no multicollinearity between independent variables in all models. Interpretation of explanatory power of all regression models followed Cohen's guidelines (1988), with *f* ^*2*^ ≥ 0.02 small, *f* ^*2*^ ≥ 0.15 medium, and *f* ^*2*^ ≥ 0.35 = large effect. Statistical analysis was carried out in SPSS v.27. Our data are publicly available in OSF.^38^

## 3 RESULTS

### 3.1 Participants

The total sample consisted of *N* = 1787 (50.3% female) participants. Age ranged from 18 to 89 years (*M* = 58.81, *SD* = 13.73). Mean age of women was 57.6 years (*SD* = 13.87), and of men 60 years (*SD* = 13.49). The most frequently diagnosed solid tumors were breast (*n* = 333, 18.6%) and lung cancer (*n* = 272, 15.2%). A total of 344 patients (19.3%) had a hematological cancer diagnosis. Table 1 details sample characteristics.

**TABLE 1:** Sociodemographic and clinical characteristics of the sample.

Characteristic	Total sample		Women		Men	
	*N* = 1787		*n* = 898		*n* = 889	
	*n*	%	*n*	%	*n*	%
Marital status						
Single	636	35.6	386	43	250	28.1
Married/partnered	1151	64.4	512	57	639	71.9
Children^a^	1295	72.5	656	73.1	639	71.9
Living area						
Rural	440	24.6	231	25.7	209	23.5
Urban	1347	75.4	667	74.3	680	76.5
Education level						
<12 years	999	55.9	509	56.7	490	55.1
≥12 years	788	44.1	389	43.3	399	44.9
Income						
≤1300 Euro	430	24.1	246	27.4	184	20.7
>1300 Euro	1357	75.9	652	72.6	705	79.3
Physical activity before cancer diagnosis						
0 h/week	509	28.5	260	29	249	28
1–2 h/week	585	32.7	324	36	261	29.4
>2 h/week	693	38.8	314	35	379	42.6
Cancer type						
Hematological	344	19.3	154	17.1	190	21.4
Solid tumor	1443	80.7	744	82.9	699	78.6
Breast	333	18.6	326	36.3	7	0.8
Lung	272	15.2	114	12.7	158	17.8
Head and neck	143	8.0	38	4.2	105	11.8
Pancreas	137	7.7	53	5.9	84	9.4
Colon/Rectum	110	6.2	37	4.1	73	8.2
Soft tissue	105	5.9	48	5.3	57	6.4
Brain	78	4.4	40	4.5	38	4.3
Stomach/esophagus	51	2.9	11	1.2	40	4.5
Kidney/urinary tract/bladder	50	2.8	13	1.4	37	4.2
Other solid	164	9.2	64	7.1	100	11.2
Anemia^b^						
No (female: Hb >11.9 g/dL, male: Hb >12.9 g/dL)	1083	60.6	601	66.9	482	54.2
Mild (female: Hb 11–11.9 g/dL, male: Hb 11–12.9 g/dL)	368	20.6	131	14.6	237	26.7
Moderate (Hb 8–10.9 g/dL)	320	17.9	158	17.6	162	18.2
Severe (Hb <8 g/dL)	16	0.9	8	0.9	8	0.9
Depression						
No depressive symptoms	1231	68.9	611	68	620	69.7
Some depressive symptoms	262	14.7	134	15	128	14.4
Significant depressive symptoms	294	16.5	153	17	141	15.9
Anxiety						
No anxiety symptoms	1098	61,4	483	53.8	615	69.2
Some anxiety symptoms	395	22.1	223	24.8	172	19.3
Significant anxiety symptoms	294	16.5	192	21.4	102	11.5
Pain^c^						
No pain	564	31.6	289	32.2	275	30.9
Mild pain	815	45.6	412	45.9	403	45.3
Moderate pain	283	15.8	133	14.8	150	16.9
Severe pain	125	7	64	7.1	61	6.9

^a^ Reflects the number and percentage of participants answering “yes” to this question.

^b^ For determining severity of anemia, classification levels of the WHO^64^ were used.

^c^ VAS pain scores were categorized as follows: no pain = 0; mild pain = 1–3, moderate pain = 4–6, and severe pain = 7–10 points.

### 3.2 Prevalence of fatigue

In total, 1441 (90.6%) patients experienced some level of fatigue. Mild fatigue was reported by 687 (38.4%), moderate fatigue by 489 (27.4%), and severe fatigue by 265 (14.8%) patients. Distribution of fatigue scores was independent of gender (χ^2^ (3, *N* = 1787) = 0.32, *p* = 0.96). Fatigue prevalence rates for various types of cancer are detailed in Table 2.

**TABLE 2:** Prevalence of fatigue.

Level of fatigue	Total sample		Women		Men		Hematological cancer		Breast cancer		Lung cancer		Head and neck cancer		Pancreatic cancer		Colon/rectum cancer		Soft tissue cancer		Brain cancer	
	*N* = 1787		*n* = 898		*n* = 889		*n* = 344		*n* = 333		*n* = 272		*n* = 143		*n* = 137		*n* = 110		*n* = 105		*n* = 78	
	*n*	%	*n*	%	*n*	%	*n*	%	*n*	%	*n*	%	*n*	%	*n*	%	*n*	%	*n*	%	*n*	%
No fatigue	346	19.4	175	19.5	171	19.2	72	20.9	85	25.5	52	19.1	25	17.5	13	9.5	17	15.5	20	19.0	16	20.5
Mild	687	38.4	342	38.1	345	38.8	145	42.2	131	39.3	99	36.4	60	42.0	51	37.2	42	38.2	40	38.1	30	38.5
Moderate	489	27.4	244	27.2	245	27.6	77	22.4	78	23.4	87	32.0	32	22.4	45	32.8	36	32.7	30	28.6	17	21.8
Severe	265	14.8	137	15.3	128	14.4	50	14.5	39	11.7	34	12.5	26	18.2	28	20.4	15	13.6	15	14.3	15	19.2

### 3.3 Relation of fatigue with psychosocial and somatic indicators

In bivariate correlation analysis, fatigue was strongly associated with pain (*r* = 0.50), depression (*r* = 0.44), and anxiety (*r* = 0.33), moderately associated with Hb levels (*r* = −0.21), and weakly associated with CRP levels (*r* = 0.18). Bivariate correlation results are detailed in Table 3.

**TABLE 3:** Bivariate correlations between key variables.

Variable	1	2	3	4	5	6	7	8	9	10	11	12	13	14	15	16	17	18	19
1. Fatigue	–																		
2. Pain	**0.498**	–																	
3. Anxiety	**0.329**	**0.316**	–																
4. Depression	**0.440**	**0.372**	**0.651**	–															
5. Hemoglobin	**−0.211**	**−0.142**	−0.029	**−0.106**	–														
6. C‐reactive protein	**0.182**	**0.244**	0.045	**0.145**	**−0.290**	–													
7. Serum creatinine	0.025	0.039	−0.024	0.066	−0.083	0.039	–												
8. Leukocyte count	−0.019	−0.001	−0.025	−0.003	−0.024	0.057	0.038	–											
9. ASAT	0.093	0.080	0.038	0.074	−0.041	**0.166**	0.043	0.004	–										
10. ALAT	0.034	0.001	0.021	0.068	0.001	0.081	−0.010	0.015	**0.642**	–									
11. Age	0.049	0.038	−0.098	0.092	−0.089	0.097	**0.219**	0.019	0.018	−0.058	–								
12. Gender	0.019	−0.010	**0.178**	0.017	**−0.121**	**−0.125**	**−0.260**	−0.081	−0.021	−0.079	−0.087	–							
13. Cancer type	−0.035	−0.074	**−0.108**	−0.095	0.017	**−0.125**	0.054	**0.129**	−0.065	−0.021	−0.070	−0.054	–						
14. Marital status	−0.032	−0.014	0.001	−0.040	0.011	0.030	0.056	−0.022	0.040	0.039	0.045	**−0.155**	−0.017	–					
15. Children	−0.035	0.011	0.013	0.024	0.021	0.007	0.031	−0.027	0.039	0.013	**0.213**	0.013	−0.017	**0.248**	–				
16. Living area	0.028	0.030	0.008	0.020	0.057	0.024	0.009	0.007	0.016	−0.007	0.090	−0.026	−0.041	**−0.118**	−0.059	–			
17. Level of education	0.013	−0.095	−0.03	−0.098	0.028	−0.084	−0.051	−0.018	−0.002	0.001	**−0.141**	−0.016	0.072	0.027	−0.066	0.079	–		
18. Income	−0.065	**−0.107**	−0.09	**−0.117**	0.067	−0.041	0.053	0.031	−0.024	0.005	−0.021	−0.078	**0.119**	**0.282**	0.028	−0.060	**0.218**	–	
19. Physical activity: 1‐2 h/week vs. 0 h/week	−0.033	−0.043	−0.021	−0.048	0.003	−0.020	−0.030	−0.007	−0.023	−0.049	**−0.106**	0.072	−0.002	0.008	−0.040	−0.014	0.053	0.030	–
20. Physical activity: >2 h/week vs. 0 h/week	−0.052	−0.022	−0.043	−0.079	0.011	−0.027	0.038	0.019	0.048	0.042	0.003	−0.079	**0.115**	0.045	−0.019	−0.020	**0.130**	**0.128**	**−0.555**

*Note*: Depending on the type of variable, Pearson's correlation coefficient, point‐biserial correlation coefficient, or phi coefficient was used. Fatigue scores were log(x + 1) transformed. Correlations showing at least a small effect (*r* ≥ 0.1) are highlighted in bold.

Abbreviations: ASAT: Aspartate aminotransferase, ALAT: Alanine aminotransferase.

Multivariate analysis using a linear regression model in the total sample revealed a significant association with pain, depression, Hb levels, having children, education level, and physical activity prior to cancer diagnosis on fatigue. Higher fatigue scores were associated with more pain, increased levels of depressive symptoms, and lower Hb levels. People with children, with lower education, and people who engaged in regular physical activity for more than 2 h per week prior to their cancer diagnosis were less likely to show symptoms of fatigue.

Separate linear regression analysis for women and men, as well as for patients with different cancer types (solid tumor and hematological cancer) showed that pain, depression, Hb levels, and education level were not significant associated with fatigue in all four subsamples. Physical activity was only significant in the male subsample and in patients with hematological cancer, while having children was only significant in patients with hematological cancer. An overview of all models tested is provided in Table 4. Tables S1 and S2 depict complete regression analysis results for all models.

**TABLE 4:** Linear regression models investigating factors associated with fatigue.

Variable	Model I: total sample		Model II: women		Model III: men		Model IV: hematologic		Model V: solid tumor	
	Estimate	*p*	Estimate	*p*	Estimate	*p*	Estimate	*p*	Estimate	*p*
Intercept	0.505	< 0.001	0.525	< 0.001	0.513	< 0.001	0.629	< 0.001	0.483	< 0.001
Pain	0.051	**< 0.001**	0.051	**< 0.001**	0.052	**< 0.001**	0.047	**< 0.001**	0.053	**< 0.001**
Anxiety	0.003	0.116	0.003	0.235	0.003	0.338	0.008	0.107	0.002	0.371
Depression	0.019	**< 0.001**	0.018	**< 0.001**	0.020	**< 0.001**	0.026	**< 0.001**	0.017	**< 0.001**
Hb levels	−0.021	**< 0.001**	−0.025	**< 0.001**	−0.018	**< 0.001**	−0.024	**< 0.001**	−0.020	**< 0.001**
CRP levels	0.002	0.411	0.004	0.232	< 0.001	0.901	−0.009	0.168	0.002	0.337
Creatinine	−0.017	0.309	−0.027	0.457	−0.014	0.458	−0.010	0.823	−0.015	0.402
Leukocytes	−0.001	0.194	−0.001	0.706	−0.001	0.213	−0.001	0.139	0.001	0.597
ASAT/GOT	<0.001	0.052	0.001	0.076	< 0.001	0.359	0.001	0.408	< 0.001	0.060
ALAT/GPT	< 0.001	0.528	2164E‐07	0.999	< 0.001	0.370	< 0.001	0.463	< 0.001	0.515
Age	0.001	0.237	0.001	0.248	< 0.001	0.768	< 0.001	0.710	0.001	0.231
Gender^a^	−0.004	0.779	–	–	–	–	−0.019	0.529	0.002	0.914
Cancer type^b^	0.028	0.093	0.033	0.165	0.022	0.353	–	–	–	–
Marital status^c^	−0.006	0.668	0.006	0.755	−0.017	0.442	−0.007	0.831	−0.003	0.852
Children^d^	−0.032	**0.030**	−0.032	0.127	−0.031	0.149	−0.087	**0.010**	−0.020	0.218
Living area^e^	0.004	0.781	0.010	0.611	−0.005	0.801	−0.030	0.348	0.009	0.584
Level of education^f^	0.055	**< 0.001**	0.038	**0.045**	0.073	**< 0.001**	0.101	**0.001**	0.047	**0.002**
Income^g^	0.009	0.561	7337E‐05	0.997	0.020	0.414	0.018	0.681	0.010	0.561
Physical activity 1–2 h/week^h^	−0.027	0.096	−0.018	0.426	−0.033	0.173	−0.006	0.880	−0.030	0.092
Physical activity >2 h/week^i^	−0.039	**0.014**	−0.032	0.174	−0.049	**0.032**	−0.042	0.302	−0.037	**0.039**
*R* ^*2*^/*R* ^*2*^ adjusted	0.35/0.35		0.37/0.35		0.35/0.33		0.43/0.40		0.34/0.34	
*f* ^*2*^	0.54		0.54		0.49		0.67		0.52	
*p*	< 0.001		< 0.001		< 0.001		< 0.001		< 0.001	
*N*	1787		898		889		344		1443	

*Note*: Values of the fatigue scale were log(x + 1) transformed due to the high skewness in fatigue scores. *f* ^*2*^: effect size interpretation according to Cohen^36^: *f* ^*2*^ ≥ 0.02 small, *f* ^*2*^ ≥ 0.15 medium, *f* ^*2*^ ≥ 0.35 = large effect.

Bold indicates significance of *p* < 0.05.

^a^ 0 = male, 1 = female.

^b^ 0 = solid tumor, 1 = hematologic cancer.

^c^ 0 = single, 1 = in partnership.

^d^ 0 = no, 1 = yes.

^e^ 0 = rural, 1 = urban.

^f^ 0 < 12 years education, 1 > 12 years education.

^g^ 0 < 1300 Euro, 1 > 1300 Euro.

^h^ 0 = no physical activity, 1 = 1‐2 h/week physical activity.

^i^ 0 = no physical activity, 1 = >2 h/week physical activity.

Explanatory power was good for all models with adjusted R^2^ ranging between 0.33 and 0.40. According to Cohen's guidelines,^36^ this represents large effects (*f* ^*2*^ ranging from 0.49 to 0.67).

## 4 DISCUSSION

Acknowledging the multifactorial etiology of fatigue, the present study examined various psychosocial and somatic factors to determine their independent association with fatigue. In the entire sample, elevated fatigue levels were linked to lower Hb levels, and increased levels of pain and depression, while lower fatigue levels were associated with lower education, regular physical activity prior to cancer diagnosis, and having children. Notably, separate analyses for men and women, and for patients with different types of cancer, revealed different relevant factors associated with CRF, supporting a personalized medicine approach. In all five tested models, pain, depression, Hb, and education were significantly related to fatigue, indicating a robust association with fatigue across the entire sample and four subsamples.

High prevalence rates of CRF across all subsamples and the overall sample were found. Over 90% of participants reported some level of fatigue, which was higher than the average results found in a recent meta‐analysis,^9^ but similar to prevalence rates reported in studies on leukemia patients.^39^ This high prevalence of fatigue indicates a high burden in cancer patients and highlights the importance of interventions targeting CRF.

Our results align with numerous studies identifying pain, depression, and Hb levels as important correlates of CRF.^26^, ^39^ As shown in recent studies focusing on clustering of symptoms, high classes are often characterized by the co‐occurrence of high levels of pain, depression, and fatigue.^2^, ^40^ In addition, patients with a high probability of pain, depression, and fatigue seem to be more prone to having lower Hb levels.^15^ Reduced Hb levels are associated with several health problems, such as breathing difficulties, headaches, sleep problems, and concentration difficulties, which have a severe impact on the patients' quality of life. New treatments for cancer addressing anemia in its early stages have demonstrated encouraging results: patients experienced decreased fatigue symptoms and improved quality of life.^41^ It should be noted, however, that the present analysis identified independent effects of Hb level, depression, and pain, implying that targeting anemia alone is not sufficient to successfully treat fatigue. Effective pain management and depression treatment should be equally considered, especially since depression is highly prevalent in oncology patients^42^ and has a negative impact on overall survival.^43^, ^44^ Moreover, elevated pain, fatigue, and depression levels are likely to result in an overall increase of symptom burden in oncological patients.^2^ In order to avoid this, targeting pain and depression, along with treating anemia, appears indispensable considering their independent association with fatigue demonstrated in this study.

Another important finding of our study was that individuals with lower education were more likely to exhibit lower fatigue, regardless of their gender and cancer type. This result is surprising since lower education has previously been associated with higher levels of CRF,^45^ as well as with more health problems^46^, ^47^ and poorer access to health care services in different samples.^48^ However, a positive relationship between CRF and education has been documented in some studies on cancer patients,^28^, ^29^ with job and social status being suggested as factors that might influence this relationship.^49^ Furthermore, the possible influence of stress, pressure, and sense of responsibility cannot be ruled out. Individuals with higher education tend to earn more and to hold executive positions with supervisor responsibilities, resulting in more pressure, stress, and difficulties in managing work‐family balance.^50^ This, in turn, might contribute to increased levels of fatigue in highly educated individuals, as shown in our study. Since this explanation is largely speculative, future studies should assess different job‐related factors (e.g., type of job, job‐related stress, and work‐family balance) in order to clarify the relationship between education, income, and CRF.

In the overall sample, as well as in patients with hematological cancer, participants with children were less likely to show symptoms of fatigue. This could be partly explained by the well‐known beneficial effect of social relations on mental health. People with children also experience more daily joy,^51^ which may buffer the negative effects of cancer, cancer treatment, and fatigue. However, longitudinal study conducted in the USA revealed a reverse effect, showing that having children has a negative impact on CRF.^6^ Later investigations in the USA confirmed this finding, particularly emphasizing the association between evening fatigue and childcare responsibilities.^17^, ^18^, ^29^ At this point, it has to be noted that a cancer diagnosis in the USA can greatly affect the financial situation of a family,^52^, ^53^ especially if there are children to care for.^54^ In Austria, every person, even those who are not currently employed, receives health insurance that covers cancer treatment, which may reduce families' financial burden. The inconsistency between the Austrian and American results could thus also be attributed to differences in the insurance policies of these countries.^55^, ^56^

According to our results, physical activity prior to cancer diagnosis was associated with fatigue only in the overall sample and in the subsample of male participants. Physical activity remains a crucial part of several interventions aiming to reduce fatigue during cancer treatment.^19^ The significant association with physical activity in men, but not in women, could be due a higher percentage of men (42.6% vs. 35%) engaging in physical activity for more than 2 h/week. In women, the most commonly reported frequency of physical activity was 1–2 h/week (36%), implying that physical activity should be performed for more than 2 h/week in order to have a beneficial effect on CRF.

The remaining psychosocial factors (age, gender, living area, relationship status, and income) and somatic factors (CRP, creatinine, leucocyte, ALAT, and ASAT) were not associated with fatigue in any of the tested models. This indicates that although these factors frequently correlate with fatigue, they do not have a significant independent association with fatigue, provided that other factors are controlled for. The association with some of these factors has already been questioned in previous investigations. For instance, female gender has been linked to CRF in many studies.^9^, ^57^, ^58^ However, a large‐scale international study could not show any differences in fatigue levels between male and female patients.^59^ Besides, a recent study on long‐term cancer survivors found fatigue to be more common in men than in women, when adjusted for age, comorbidities, partner, and employment status.^60^ Thus, higher levels of CRF in women might arise if important covariates are not adjusted for. This could explain why no gender effects could be detected in our study, which controlled for numerous psychosocial and somatic factors. The gender‐related inconsistencies in literature may also be due to heterogeneous samples, as proposed in an earlier study.^61^ Apart from gender, age is another often disputed risk factor for CRF. Some studies have linked younger age to increased levels of fatigue,^2^ while others showed higher levels of fatigue in older patients.^62^ According to a study on mixed‐diagnosis cancer samples, the latter could be partly explained by Hb levels.^63^ Notably, a systematic review including studies published between 1995 and 2020 did not find an association between age and fatigue.^9^ In this review, the significant association observed in some studies was attributed to more advanced stages of treatment.

Taken together, there are several possible explanations for the discrepancies related to age and gender differences in CRF. Age and gender, but also other factors that were not related to fatigue in our study, might be a result of a high interindividual variability in fatigue levels found in previous investigations.^18^ To address this variability, personalized medicine approach is recommended both in research and clinical practice.

### 4.1 Strengths and limitations

The major strengths of the present study are the inclusion of a large number of psychosocial and somatic indicators, as well as the determination of their unique contribution to CRF in different subsamples. In addition to patients with solid tumors, we also included patients with hematological cancer, that have been comparably less studied. Furthermore, the sample was sufficiently large and well‐balanced in terms of sociodemographic characteristics and cancer types. One limitation of the current study relates to the clinical characteristics of the sample. We did not assess information on patients' treatment history, time of diagnosis, cancer stage, and prognosis, which limits the interpretation of the present results. We were also unable to control for some CRF‐related aspects, such as smoking status, due to lack of data. However, our sample is a real‐life mixed cancer sample, as encountered in a large public outpatient clinic, and therefore has high external validity.

Considering multiple possible determinants, the study has provided new insights into factors associated with CRF, but it also has some limitations. For both fatigue and pain, we used one‐item screenings. Although both measures have good psychometric properties and are widely used, they do not allow for a differentiated assessment of fatigue and pain and cannot capture a multidimensional conceptualization (i.e., comprising physical, emotional, and cognitive dimensions). Different indicators may have been associated with different dimensions of fatigue. Further differentiation regarding income and education level is also warranted in upcoming studies, since we only considered two levels, respectively. Additional information about the type of physical activity would also be beneficial to better understand the underlying physiological mechanisms that could improve fatigue prevention and intervention programs.

### 4.2 Conclusions

Our study provides insights in the multifactorial pathogenesis of fatigue, comprising various biopsychosocial correlates, all of which contribute differently to CRF depending on gender and cancer type. While certain indicators such as pain, depression, education, and a lover level of Hb appear to be universally relevant, physical activity and parenthood exhibit positive effects only in some target groups. Notably, many of the identified factors associated with CRF are modifiable, which has important implications for fatigue research and practice, including fatigue treatment. When treating CRF, people with higher education should be given extra attention, as they seem to be confronted with specific challenges leading to higher levels of fatigue and potentially impact the recovery process. Moreover, the management of Hb levels and pain, along with the treatment of depression, should be an indispensable component of cancer treatment, with promising effects on reducing fatigue and improving quality of life in cancer patients.

## AUTHOR CONTRIBUTIONS

**Elisabeth L. Zeilinger:** Conceptualization (equal); data curation (lead); formal analysis (lead); investigation (lead); methodology (equal); project administration (lead); validation (equal); writing – original draft (lead). **Irina Zrnic‐Novakovic:** Formal analysis (supporting); validation (equal); writing – original draft (supporting). **Claudia Oppenauer:** Formal analysis (supporting); investigation (supporting); writing – original draft (supporting). **Matthäus Fellinger:** Formal analysis (supporting); writing – original draft (supporting). **Matthias Knefel:** Investigation (supporting); writing – review and editing (equal). **Matthias Unseld:** Investigation (supporting); writing – review and editing (equal). **Theresa Wagner:** Investigation (supporting); writing – review and editing (equal). **Simone Lubowitzki:** Investigation (supporting); writing – review and editing (equal). **Rupert Bartsch:** Investigation (supporting); writing – review and editing (equal). **Sabine Zöchbauer‐Müller:** Investigation (supporting); writing – review and editing (equal). **Markus Raderer:** Investigation (supporting); writing – review and editing (equal). **Philipp B. Staber:** Investigation (supporting); writing – review and editing (equal). **Peter Valent:** Investigation (supporting); writing – review and editing (equal). **Alexander Gaiger:** Conceptualization (equal); methodology (equal); supervision (lead); writing – review and editing (lead).

## FUNDING INFORMATION

This research did not receive any specific grant from funding agencies in the public, commercial, or not‐for‐profit sectors.

## CONFLICT OF INTEREST STATEMENT

Rupert Bartsch: Consulting fees: Astra‐Zeneca, Daiichi, Eisai, Eli‐Lilly, Gilead, Gruenenthal, MSD, Novartis, Pfizer, Pierre‐Fabre, Puma, Roche, Seagen. Lecture Honoraria: Astra‐Zeneca, Daichi, Eisai, Eli‐Lilly, Gilead, Gruenenthal, MSD, Novartis, Pfizer, Pierre‐Fabre, Roche, Seagen. Support for attending meetings and/or travel: AstraZeneca, Daiichi, MSD, Roche. Peter Valent: Consulting fees: Novartis, Blueprint, BMS/Celgene, Pfizer, Cogent, and AOP Orphan. All other authors have nothing to disclose.

## Supporting information


Table S1.

Table S2.

## Data Availability

The data that support the findings of this study are openly available in OSF at https://doi.org/10.17605/OSF.IO/YHF98.

## References

[1] Berger AM , Mooney K , Alvarez‐Perez A , et al. Cancer‐related fatigue, version 2.2015. J Natl Compr Cancer Netw. 2015;13(8):1012‐1039. doi:10.6004/jnccn.2015.0122 PMC5499710 26285247

[2] Hammer MJ , Cooper B , Paul SM , et al. Identification of distinct symptom profiles in cancer patients using a pre‐specified symptom cluster. J Pain Symptom Manag. 2022;64(1):17‐27. doi:10.1016/j.jpainsymman.2022.03.007 35339613

[3] IJsbrandy C , Ottevanger PB , Gerritsen WR , van Harten WH , Hermens RPMG . Determinants of adherence to physical cancer rehabilitation guidelines among cancer patients and cancer centers: a cross‐sectional observational study. J Cancer Surviv. 2021;15(1):163‐177. doi:10.1007/s11764-020-00921-8 32986232 PMC7822788

[4] Weber D , O'Brien K . Cancer and cancer‐related fatigue and the interrelationships with depression, stress, and inflammation. J Evid Based Complementary Altern Med. 2017;22(3):502‐512. doi:10.1177/2156587216676122 30208733 PMC5871160

[5] Knefel M , Zeilinger EL , Erfurth A , et al. Affective temperament, fatigue, and pain in cancer patients. J Affect Disord. 2023;340:80‐87. doi:10.1016/j.jad.2023.08.003 37543112

[6] Dhruva A , Dodd M , Paul SM , et al. Trajectories of fatigue in patients with breast cancer before, during, and after radiation therapy. Cancer Nurs. 2010;33(3):201‐212. doi:10.1097/NCC.0b013e3181c75f2a 20357659 PMC2881569

[7] Manitta V , Zordan R , Cole‐Sinclair M , Nandurkar H , Philip J . The symptom burden of patients with hematological malignancy: a cross‐sectional observational study. J Pain Symptom Manag. 2011;42(3):432‐442. doi:10.1016/j.jpainsymman.2010.12.008 21477979

[8] Lacourt TE , Kavelaars A , Ohanian M , et al. Patient‐reported fatigue prior to treatment is prognostic of survival in patients with acute myeloid leukemia. Oncotarget. 2018;9(58):31244‐31252. doi:10.18632/oncotarget.25787 30131851 PMC6101294

[9] Al Maqbali M , Al Sinani M , Al Naamani Z , Al Badi K , Tanash MI . Prevalence of fatigue in patients with cancer: a systematic review and meta‐analysis. J Pain Symptom Manag. 2021;61(1):167‐189.e14. doi:10.1016/j.jpainsymman.2020.07.037 32768552

[10] Kuhnt S , Friedrich M , Schulte T , Esser P , Hinz T . Predictors of fatigue in cancer patients: a longitudinal study. Support Care Cancer. 2019;27(9):3463‐3471. doi:10.1007/s00520-019-4660-4 30680616

[11] Bower JE . The role of neuro‐immune interactions in cancer‐related fatigue: biobehavioral risk factors and mechanisms. Cancer. 2019;125(3):353‐364. doi:10.1002/cncr.31790 30602059 PMC6502236

[12] Feng LR , Barb JJ , Regan J , Saligan LN . Plasma metabolomic profile associated with fatigue in cancer patients. Cancer Med. 2021;10(5):1623‐1633. doi:10.1002/cam4.3749 33534943 PMC7940245

[13] Saligan LN , Olson K , Filler K , et al. The biology of cancer‐related fatigue: a review of the literature. Support Care Cancer. 2015;23(8):2461‐2478. doi:10.1007/s00520-015-2763-0 25975676 PMC4484308

[14] Goklemez S , Saligan LN , Pirsl F , et al. Clinical characterization and cytokine profile of fatigue in hematologic malignancy patients with chronic graft‐versus‐host disease. Bone Marrow Transplant. 2021;56(12):2934‐2939. doi:10.1038/s41409-021-01419-2 34433916 PMC8639672

[15] Jhamb M , Abdel‐Kader K , Yabes J , et al. Comparison of fatigue, pain, and depression in patients with advanced kidney disease and cancer ‐ symptom burden and clusters. J Pain Symptom Manag. 2019;57(3):566‐575.e3. doi:10.1016/j.jpainsymman.2018.12.006 PMC6382584 30552961

[16] McCall MK , Connolly M , Nugent B , Conley YP , Bender CM , Rosenzweig MQ . Symptom experience, management, and outcomes according to race and social determinants including genomics, epigenomics, and metabolomics (SEMOARS + GEM): an explanatory model for breast cancer treatment disparity. J Cancer Educ. 2020;35(3):428‐440. doi:10.1007/s13187-019-01571-w 31392599 PMC7245588

[17] Dhruva A , Aouizerat BE , Cooper B , et al. Cytokine gene associations with self‐report ratings of morning and evening fatigue in oncology patients and their family caregivers. Biol Res Nurs. 2015;17(2):175‐184. doi:10.1177/1099800414534313 24872120 PMC5486216

[18] Wright F , D'Eramo Melkus G , Hammer M , et al. Predictors and trajectories of morning fatigue are distinct from evening fatigue. J Pain Symptom Manag. 2015;50(2):176‐189. doi:10.1016/j.jpainsymman.2015.02.016 PMC4526314 25828559

[19] Goedendorp MM , Gielissen MF , Verhagen CA , Bleijenberg G . Psychosocial interventions for reducing fatigue during cancer treatment in adults. Cochrane Database Syst Rev. 2009;2009(1):CD006953. doi:10.1002/14651858.CD006953.pub2 19160308 PMC7197415

[20] Cedenilla Ramón N , Calvo Arenillas JI , Aranda Valero S , Sánchez Guzmán A , Moruno MP . Psychosocial interventions for the treatment of cancer‐related fatigue: an umbrella review. Curr Oncol. 2023;30(3):2954‐2977. doi:10.3390/curroncol30030226 36975439 PMC10047125

[21] Belloni S , Arrigoni C , Caruso R . Effects from physical exercise on reduced cancer‐related fatigue: a systematic review of systematic reviews and meta‐analysis. Acta Oncol. 2021;60(12):1678‐1687. doi:10.1080/0284186X.2021.1962543 34396915

[22] Bower JE , Wiley J , Petersen L , Irwin MR , Cole SW , Ganz PA . Fatigue after breast cancer treatment: biobehavioral predictors of fatigue trajectories. Health Psychol. 2018;37:1025‐1034. doi:10.1037/hea0000652 30321021 PMC6372460

[23] Jakovljevic K , Kober KM , Block A , et al. Higher levels of stress are associated with a significant symptom burden in oncology outpatients receiving chemotherapy. J Pain Symptom Manag. 2021;61(1):24‐31.e4. doi:10.1016/j.jpainsymman.2020.07.019 PMC7770050 32721501

[24] Bower JE , Ganz PA , Irwin MR , et al. Do all patients with cancer experience fatigue? A longitudinal study of fatigue trajectories in women with breast cancer. Cancer. 2021;127(8):1334‐1344. doi:10.1002/cncr.33327 33606273 PMC8562726

[25] Unseld M , Zeilinger EL , Fellinger M , et al. Prevalence of pain and its association with symptoms of post‐traumatic stress disorder, depression, anxiety and distress in 846 cancer patients: a cross sectional study. Psycho‐Oncol. 2021;30(4):504‐510. doi:10.1002/pon.5595 PMC8049050 33210393

[26] Hajj A , Chamoun R , Salameh P , et al. Fatigue in breast cancer patients on chemotherapy: a cross‐sectional study exploring clinical, biological, and genetic factors. BMC Cancer. 2022;22(1):16. doi:10.1186/s12885-021-09072-0 34979978 PMC8722263

[27] Mao H , Bao T , Shen X , et al. Prevalence and risk factors for fatigue among breast cancer survivors on aromatase inhibitors. Eur J Cancer. 2018;101:47‐54. doi:10.1016/j.ejca.2018.06.009 30014974 PMC6148367

[28] Huang HP , Chen ML , Liang J , Miaskowski C . Changes in and predictors of severity of fatigue in women with breast cancer: a longitudinal study. Int J Nurs Stud. 2014;51(4):582‐592. doi:10.1016/j.ijnurstu.2013.09.003 24094610

[29] Wright F , D'Eramo Melkus G , Hammer M , et al. Trajectories of evening fatigue in oncology outpatients receiving chemotherapy. J Pain Symptom Manag. 2015;50(2):163‐175. doi:10.1016/j.jpainsymman.2015.02.015 PMC4526403 25828560

[30] Cramer H , Lauche R , Klose P , Lange S , Langhorst J , Dobos GJ . Yoga for improving health‐related quality of life, mental health and cancer‐related symptoms in women diagnosed with breast cancer. Cochrane Database Syst Rev. 2017;1:CD010802. doi:10.1002/14651858.CD010802.pub2 28045199 PMC6465041

[31] Temel JS , Pirl WF , Recklitis CJ , Cashavelly B , Lynch TJ . Feasibility and validity of a one‐item fatigue screen in a thoracic oncology clinic. J Thorac Oncol. 2006;1(5):454‐459. doi:10.1016/S1556-0864(15)31611-7 17409899

[32] Breivik H , Borchgrevink PC , Allen SM , et al. Assessment of pain. Br J Anaesth. 2008;101(1):17‐24. doi:10.1093/bja/aen103 18487245

[33] Snaith RP . The hospital anxiety and depression scale. Health Qual Life Outcomes. 2003;1:29. doi:10.1186/1477-7525-1-29 12914662 PMC183845

[34] Zeilinger EL , Nader IW , Wiedermann W , et al. Latent structure and measurement invariance of the hospital anxiety and depression scale in cancer outpatients. Int J Clin Health Psychol. 2022;22(3):100315. doi:10.1016/j.ijchp.2022.100315 35662789 PMC9157192

[35] Hammouri HM , Sabo RT , Alsaadawi R , Kheirallah KA . Handling skewed data: a comparison of two popular methods. Appl Sci. 2020;10(18):6247. doi:10.3390/app10186247

[36] Cohen J . Statistical Power Analysis for the Behavioral Sciences. 2nd ed. Lawrence Erlbaum Associates; 1988.

[37] Gignac GE , Szodorai ET . Effect size guidelines for individual differences researchers. Personal Individ Differ. 2016;102:74‐78. doi:10.1016/j.paid.2016.06.069

[38] Zeilinger EL , Gaiger A . Data from: fatigue in cancer outpatients. *OSF* . 2022. doi:10.17605/OSF.IO/YHF98

[39] Musarezaie A , Khaledi F , Esfahani HN , Ghaleghasemi TM . Factors affecting quality of life and fatigue in patients with leukemia under chemotherapy. J Educ Health Promot. 2014;3(64):74‐79. 25077157 10.4103/2277-9531.134778 PMC4113984

[40] Shin J , Harris C , Oppegaard K , et al. Worst pain severity Profiles of oncology patients are associated with significant stress and multiple co‐occurring symptoms. J Pain. 2022;23(1):74‐88. doi:10.1016/j.jpain.2021.07.001 34298161 PMC10788964

[41] Bekes I , Eichler M , Singer S , et al. Impact of granulocyte colony‐stimulating factor (G‐CSF) and epoetin (EPO) on hematologic toxicities and quality of life in patients during adjuvant chemotherapy in early breast cancer: results from the multi‐center randomized ADEBAR trial. Clin Breast Cancer. 2020;20(6):439‐447. doi:10.1016/j.clbc.2020.03.008 32800493

[42] Zeilinger EL , Oppenauer C , Knefel M , et al. Prevalence of anxiety and depression in people with different types of cancer or haematologic malignancies: a cross‐sectional study. Epidemiol Psychiatr Sci. 2022;31:e74. doi:10.1017/S2045796022000592 36245424 PMC9583630

[43] Gaiger A , Lubowitzki S , Krammer K , et al. The cancer survival index—a prognostic score integrating psychosocial and biological factors in patients diagnosed with cancer or haematologic malignancies. Cancer Med. 2022;11(18):3387‐3396. doi:10.1002/cam4.4697 35315594 PMC9487871

[44] Knefel M , Zeilinger EL , Lubowitzki S , et al. Risk as a pattern over time: delineation of time‐dependent risk factors in biological, psychological, and social variables in cancer patients. Cancer. 2023;129(21):3466‐3475. doi:10.1002/cncr.34953 37470252

[45] Andrykowski MA , Donovan KA , Jacobsen PB . Magnitude and correlates of response shift in fatigue ratings in women undergoing adjuvant therapy for breast cancer. J Pain Symptom Manag. 2009;37(3):341‐351. doi:10.1016/j.jpainsymman.2008.03.015 PMC2682229 18757176

[46] Raghupathi V , Raghupathi W . The influence of education on health: an empirical assessment of OECD countries for the period 1995–2015. Arch Public Health. 2020;78(1):20. doi:10.1186/s13690-020-00402-5 32280462 PMC7133023

[47] Zeilinger EL , Knefel M , Schneckenreiter C , et al. The impact of COVID‐19 and socioeconomic status on psychological distress in cancer patients. Int J Clin Health Psychol. 2023;23(4):100404. doi:10.1016/j.ijchp.2023.100404 37663044 PMC10469068

[48] Zeilinger EL , Lubowitzki S , Unseld M , et al. The impact of COVID‐19 on cancer care of outpatients with low socioeconomic status. Int J Cancer. 2022;17:77‐82. doi:10.1002/ijc.33960 PMC9087749 35128650

[49] Akechi T , Kugaya A , Okamura H , Yamawaki S , Uchitomi Y . Fatigue and its associated factors in ambulatory cancer patients. J Pain Symptom Manag. 1999;17(1):42‐48. doi:10.1016/S0885-3924(98)00105-5 9919864

[50] Schieman S , Glavin P . Education and work‐family Conflict: explanations, contingencies and mental health consequences. Soc Forces. 2011;89(4):1341‐1362. doi:10.1093/sf/89.4.1341

[51] Deaton A , Stone AA . Evaluative and hedonic wellbeing among those with and without children at home. Proc Natl Acad Sci USA. 2014;111(4):1328‐1333. doi:10.1073/pnas.1311600111 24474755 PMC3910586

[52] Yabroff KR , Bradley C , Shih YCT . Understanding financial hardship among cancer survivors in the United States: strategies for prevention and mitigation. J Clin Oncol. 2020;38(4):292‐301. doi:10.1200/JCO.19.01564 31804869 PMC6994250

[53] Han X , Zhao J , Zheng Z , De Moor JS , Virgo KS , Yabroff KR . Medical financial hardship intensity and financial sacrifice associated with cancer in the United States. Cancer Epidemiol Biomarkers Prev. 2020;29(2):308‐317. doi:10.1158/1055-9965.EPI-19-0460 31941708 PMC7007367

[54] Richard P . The burden of medical debt faced by households with dependent children in the United States: implications for the affordable care act of 2010. J Fam Econ Iss. 2016;37(2):212‐225. doi:10.1007/s10834-016-9491-6

[55] Aggarwal A , Ginsburg O , Fojo T . Cancer economics, policy and politics: what informs the debate? Perspectives from the EU, Canada and US. J Cancer Policy. 2014;2(1):1‐11. doi:10.1016/j.jcpo.2014.02.002

[56] Sullivan R , Peppercorn J , Sikora K , et al. Delivering affordable cancer care in high‐income countries. Lancet Oncol. 2011;12(10):933‐980. doi:10.1016/S1470-2045(11)70141-3 21958503

[57] Wang Y , Tian L , Liu X , et al. Multidimensional predictors of cancer‐related fatigue based on the predisposing, precipitating, and perpetuating (3P) model: a systematic review. Cancer. 2023;15(24):5879. doi:10.3390/cancers15245879 PMC10741552 38136423

[58] Kang YE , Yoon JH , Park NH , Ahn YC , Lee EJ , Son CG. Prevalence of cancer‐related fatigue based on severity: a systematic review and meta‐analysis. Sci Rep 2023;13(1): 12815. doi:10.1038/s41598-023-39046-0 37550326 PMC10406927

[59] Koch M , Hjermstad MJ , Tomaszewski K , et al. Gender effects on quality of life and symptom burden in patients with lung cancer: results from a prospective, cross‐cultural, multi‐center study. J Thorac Dis. 2020;12(8):4253‐4261. doi:10.21037/jtd-20-1054 32944337 PMC7475557

[60] Oertelt‐Prigione S , de Rooij BH , Mols F , et al. Sex‐differences in symptoms and functioning in >5000 cancer survivors: results from the PROFILES registry. Eur J Cancer. 2021;156:24‐34. doi:10.1016/j.ejca.2021.07.019 34411849

[61] Miaskowski C . Gender differences in pain, fatigue, and depression in patients with cancer. J Natl Cancer Inst Monogr. 2004;32:139‐143. doi:10.1093/jncimonographs/lgh024 15263057

[62] Fleer J , Sleijfer DT , Hoekstra HJ , Tuinman MA , Hoekstra‐Weebers JEHM . Prevalence, changes in and correlates of fatigue in the first year after diagnosis of testicular cancer. Anticancer Res. 2005;25(6C):4647‐4653. 16334156

[63] Butt Z , Rao AV , Lai JS , Abernethy AP , Rosenbloom SK , Cella D . Age‐associated differences in fatigue among patients with cancer. J Pain Symptom Manag. 2010;40(2):217‐223. doi:10.1016/j.jpainsymman.2009.12.016 PMC2921482 20541901

[64] World Health Organization . Haemoglobin concentrations for the diagnosis of anaemia and assessment of severity. 2011 https://apps.who.int/iris/handle/10665/85839

