# Supplementary material for: Prevalence and biopsychosocial indicators of fatigue in cancer patients
Source: Cancer Med. 2024 May 31;13(11):e7293. doi: 10.1002/cam4.7293 (PMC11141333; doi:10.1002/cam4.7293)
Supplement: Supplementary file 1 — Table S1. [file CAM4-13-e7293-s001.docx]

**Supplementary Table 1.** Linear regression models investigating *factors associated with* fatigue

| Variable | Model I: total sample | | | | |  | Model II: women | | | | |  | Model III: men | | | | |
| --- | --- | --- | --- | --- | --- | --- | --- | --- | --- | --- | --- | --- | --- | --- | --- | --- | --- |
|  | Estimate | *SE* | 95% *CI* | | *p* |  | Estimate | *SE* | 95% *CI* | | *p* |  | Estimate | *SE* | 95% *CI* | | *p* |
|  |  |  | *LL* | *UL* |  |  |  |  | *LL* | *UL* |  |  |  |  | *LL* | *UL* |  |
| Intercept | .505 | .075 | .358 | .652 | <.001 |  | .525 | .100 | .328 | .722 | <.001 |  | .513 | .095 | .327 | .698 | <.001 |
| Pain | .051 | .003 | .046 | .057 | **<.001** |  | .051 | .004 | .043 | .060 | **<.001** |  | .052 | .004 | .043 | .060 | **<.001** |
| Anxiety | .003 | .002 | -.001 | .007 | .116 |  | .003 | .003 | -.002 | .009 | .235 |  | .003 | .003 | -.003 | .009 | .338 |
| Depression | .019 | .002 | .015 | .022 | **<.001** |  | .018 | .003 | .012 | .023 | **<.001** |  | .020 | .003 | .014 | .025 | **<.001** |
| Hb levels | -.021 | .003 | -.028 | -.014 | **<.001** |  | -.025 | .006 | -.036 | -.014 | **<.001** |  | -.018 | .005 | -.027 | -.009 | **<.001** |
| CRP levels | .002 | .002 | -.002 | .005 | .411 |  | .004 | .003 | -.003 | .011 | .232 |  | <.001 | .002 | -.005 | .005 | .901 |
| Creatinine | -.017 | .016 | -.049 | .015 | .309 |  | -.027 | .036 | -.097 | .044 | .457 |  | -.014 | .019 | -.050 | .023 | .458 |
| Leukocytes | -.001 | <.001 | -.001 | <.001 | .194 |  | -.001 | .002 | -.004 | .002 | .706 |  | -.001 | <.001 | -.002 | <.001 | .213 |
| ASAT / GOT | <.001 | <.001 | <.001 | .001 | .052 |  | .001 | <.001 | <.001 | .001 | .076 |  | <.001 | <.001 | <.001 | .001 | .359 |
| ALAT / GPT | <.001 | <.001 | -.001 | <.001 | .528 |  | 2,164E-07 | <.001 | -.001 | .001 | .999 |  | <.001 | <.001 | -.001 | <.001 | .370 |
| Age | .001 | .001 | <.001 | .002 | .237 |  | .001 | .001 | -.001 | .002 | .248 |  | <.001 | .001 | -.001 | .002 | .768 |
| Gender ^a^ | -.004 | .014 | -.031 | .023 | .779 |  | - | - | - | - | - |  | - | - | - | - | - |
| Cancer type ^b^ | .028 | .016 | -.005 | .060 | .093 |  | .033 | .024 | -.014 | .080 | .165 |  | .022 | .023 | -.024 | .067 | .353 |
| Marital status ^c^ | -.006 | .014 | -.034 | .022 | .668 |  | .006 | .019 | -.031 | .043 | .755 |  | -.017 | .022 | -.061 | .027 | .442 |
| Children ^d^ | -.032 | .015 | -.061 | -.003 | **.030** |  | -.032 | .021 | -.073 | .009 | .127 |  | -.031 | .021 | -.073 | .011 | .149 |
| Living area ^e^ | .004 | .015 | -.025 | .033 | .781 |  | .010 | .021 | -.030 | .051 | .611 |  | -.005 | .022 | -.048 | .037 | .801 |
| Level of education ^f^ | .055 | .013 | .029 | .081 | **<.001** |  | .038 | .019 | .001 | .075 | **.045** |  | .073 | .019 | .036 | .110 | **<.001** |
| Income ^g^ | .009 | .016 | -.022 | .040 | .561 |  | 7,337E-05 | .021 | -.042 | .042 | .997 |  | .020 | .025 | -.028 | .069 | .414 |
| Physical activity 1-2h/week ^h^ | -.027 | .016 | -.060 | .005 | .096 |  | -.018 | .023 | -.063 | .027 | .426 |  | -.033 | .024 | -.080 | .014 | .173 |
| Physical activity >2h/week ^i^ | -.039 | .016 | -.071 | -.008 | **.014** |  | -.032 | .023 | -.077 | .014 | .174 |  | -.049 | .023 | -.093 | -.004 | **.032** |
| *R^2^* / *R^2^* adjusted | 0.35 / **0.35** | | | | |  | 0.37 / **0.35** | | | | |  | 0.35 / **0.33** | | | | |
| *f^2^* | 0.54 | | | | |  | 0.54 | | | | |  | 0.49 | | | | |
| *p* | < .001 | | | | |  | < .001 | | | | |  | < .001 | | | | |
| *N* | 1,787 | | | | |  | 898 | | | | |  | 889 | | | | |

*Note.* Values of the fatigue scale were log(x+1) transformed due to the high skewness in fatigue scores. *f^2^:* effect size interpretation according to Cohen (1988): *f^2^* ≥ 0.02 small, *f^2^* ≥ 0.15 medium, *f^2^* ≥ 0.35 = large effect.

^a^ 0 = male, 1 = female. ^b^ 0 = solid tumour, 1 = haematological cancer. ^c^ 0 = single, 1 = in partnership. ^d^ 0 = no, 1 = yes. ^e^ 0 = rural, 1 = urban. ^f^ 0 <12 years education, 1 >12 years education. ^g^ 0 < 1,300 Euro, 1 > 1,300 Euro. ^h^ 0 = no physical activity, 1 = 1-2h/week physical activity. ^i^ 0 = no physical activity, 1 = >2h/week physical activity.
