# Supplementary material for: Prevalence and biopsychosocial indicators of fatigue in cancer patients
Source: Cancer Med. 2024 May 31;13(11):e7293. doi: 10.1002/cam4.7293 (PMC11141333; doi:10.1002/cam4.7293)
Supplement: Supplementary file 2 — Table S2. [file CAM4-13-e7293-s002.docx]

**Supplementary Table 2**. Linear regression models investigating *factors associated with* fatigue

| Variable | Model IV: haematological cancer | | | | |  | Model V: solid tumour | | | | |  |
| --- | --- | --- | --- | --- | --- | --- | --- | --- | --- | --- | --- | --- |
|  | Estimate | *SE* | 95% *CI* | | *p* |  | Estimate | *SE* | 95% *CI* | | *p* |  |
|  |  |  | *LL* | *UL* |  |  |  |  | *LL* | *UL* |  |  |
| Intercept | .629 | .154 | .327 | .932 | <.001 |  | .483 | .082 | .321 | .645 | <.001 |  |
| Pain | .047 | .007 | .032 | .061 | **<.001** |  | .053 | .003 | .046 | .059 | **<.001** |  |
| Anxiety | .008 | .005 | -.002 | .017 | .107 |  | .002 | .002 | -.002 | .007 | .371 |  |
| Depression | .026 | .005 | .017 | .036 | **<.001** |  | .017 | .002 | .013 | .021 | **<.001** |  |
| Hb levels | -.024 | .007 | -.038 | -.011 | **<.001** |  | -.020 | .004 | -.028 | -.012 | **<.001** |  |
| CRP levels | -.009 | .007 | -.022 | .004 | .168 |  | .002 | .002 | -.002 | .006 | .337 |  |
| Creatinine | -.010 | .046 | -.101 | .080 | .823 |  | -.015 | .018 | -.049 | .020 | .402 |  |
| Leukocytes | -.001 | <.001 | -.002 | <.001 | .139 |  | .001 | .002 | -.002 | .004 | .597 |  |
| ASAT / GOT | .001 | .002 | -.002 | .004 | .408 |  | <.001 | <.001 | <.001 | .001 | .060 |  |
| ALAT / GPT | <.001 | .001 | -.001 | .001 | .463 |  | <.001 | <.001 | -.001 | <.001 | .515 |  |
| Age | <.001 | .001 | -.002 | .003 | .710 |  | .001 | .001 | <.001 | .002 | .231 |  |
| Gender ^a^ | -.019 | .030 | -.079 | .041 | .529 |  | .002 | .016 | -.029 | .032 | .914 |  |
| Marital status ^b^ | -.007 | .032 | -.069 | .056 | .831 |  | -.003 | .016 | -.035 | .029 | .852 |  |
| Children ^c^ | -.087 | .034 | -.154 | -.021 | **.010** |  | -.020 | .017 | -.053 | .012 | .218 |  |
| Living area ^d^ | -.030 | .032 | -.093 | .033 | .348 |  | .009 | .017 | -.024 | .042 | .584 |  |
| Level of education ^e^ | .101 | .029 | .044 | .159 | **.001** |  | .047 | .015 | .018 | .077 | **.002** |  |
| Income ^f^ | .018 | .043 | -.067 | .102 | .681 |  | .010 | .017 | -.024 | .044 | .561 |  |
| Physical activity 1-2h/week ^g^ | -.006 | .042 | -.090 | .077 | .880 |  | -.030 | .018 | -.066 | .005 | .092 |  |
| Physical activity >2h/week ^h^ | -.042 | .040 | -.121 | .038 | .302 |  | -.037 | .018 | -.072 | -.002 | **.039** |  |
| *R^2^* / *R^2^* adjusted | 0.43 / **0.40** | | | | |  | 0.34 / **0.34** | | | | |  |
| *f^2^* | 0.67 | | | | |  | 0.52 | | | | |  |
| *p* | < .001 | | | | |  | < .001 | | | | |  |
| *N* | 344 | | | | |  | 1,443 | | | | |  |

*Note.* Values of the fatigue scale were log(x+1) transformed due to the high skewness in fatigue scores. *f^2^:* effect size interpretation according to Cohen (1988): *f^2^* ≥ 0.02 small, *f^2^* ≥ 0.15 medium, *f^2^* ≥ 0.35 = large effect.

^a^ 0 = male, 1 = female. ^b^ 0 = single, 1 = in partnership. ^c^ 0 = no, 1 = yes. ^d^ 0 = rural, 1 = urban. ^e^ 0 <12 years education, 1 >12 years education. ^f^ 0 < 1,300 Euro, 1 > 1,300 Euro. ^g^ 0 = no physical activity, 1 = 1-2h/week physical activity. ^h^ 0 = no physical activity, 1 = >2h/week physical activity.
